# Encapsulation of Cinnamaldehyde and Vanillin as a Strategy to Increase Their Antimicrobial Activity

**DOI:** 10.3390/foods13132032

**Published:** 2024-06-27

**Authors:** Francisco Sepúlveda, Luis Puente-Diaz, Jaime Ortiz-Viedma, Alicia Rodríguez, Cielo Char

**Affiliations:** 1 Departamento de Ciencias de los Alimentos y Tecnología Química, Facultad de Ciencias Químicas y Farmacéuticas, Universidad de Chile, Av. Dr. Carlos Lorca Tobar 964, Independencia, Santiago P.O. Box 1004, Chile; fransepulveda@ug.uchile.cl (F.S.); lpuente@ciq.uchile.cl (L.P.-D.); jaortiz@uchile.cl (J.O.-V.); arodrigm@uchile.cl (A.R.); 2 Department of Agricultural, Food and Nutritional Science, University of Alberta, Edmonton, AB T6G 2P5, Canada

**Keywords:** whey protein, vanillin, cinnamaldehyde, *Listeria innocua*, *Escherichia coli*, *Saccharomyces cerevisiae*

## Abstract

Many studies have suggested that the encapsulation of natural antimicrobials increases their antimicrobial activity. In this sense, the objective was to study the inactivation of microorganisms with encapsulated cinnamaldehyde and vanillin (E-CIN and E-VN), in comparison with the unencapsulated antimicrobials (CIN and VN) in protein beverages. Additionally, the microbial response was quantified through mathematical modeling. Cinnamaldehyde and vanillin were encapsulated using whey protein concentrate (WPC) as the encapsulating agent. The effectiveness at inactivating *Escherichia coli*, *Listeria innocua*, and *Saccharomyces cerevisiae* was evaluated in a protein-apple juice beverage during storage (4 °C). Encapsulation increased the effectiveness of cinnamaldehyde, reaching reductions of 1.8, 3.3, and 5.3 log CFU/mL in *E. coli*, *L. innocua*, and *S. cerevisiae*, respectively, while vanillin encapsulation had little effect on antimicrobial activity, reducing by 0.5, 1.4, and 1.1 log cycles, respectively. The combined treatments (E-CIN + E-VN) had an additive effect in reducing *E. coli* and a synergistic effect against *S. cerevisiae*. The Gompertz model was more versatile and better described the biphasic curves, whereas the Weibull model complemented the information regarding the spectrum of resistances within the microbial population. In conclusion, the encapsulation of cinnamaldehyde with WPC enhanced its activity. However, further studies are necessary to improve the antimicrobial activity of vanillin.

## 1. Introduction

Chemically synthesized preservatives such as potassium sorbate and sodium benzoate have been widely used by the food industry to control microbial growth and food spoilage and to increase the shelf life of products. However, the use of these antimicrobials is controversial since it has been reported that they are possible causes of respiratory diseases and other health risks, such as asthma, hives, and suppression of the immune system [1,2,3]. For this reason, the food industry has become interested in replacing these chemical additives, considering natural antimicrobials as a healthier alternative. Furthermore, these natural antimicrobials have been considered suitable in replacing the use of chemical fungicides in the control of postharvest fruit diseases [4].

Essential oils (EOs) are natural compounds derived from plants, which are recognized as safe (GRAS) and present various degrees of antimicrobial, antifungal, and antiviral activity [5]. Among them, cinnamaldehyde (3-phenyl-2-propenal, CIN), the main component of cinnamon bark, is a hydrophobic aromatic aldehyde used as flavoring in the food industry. Multiple reports have involved CIN with a broad antibacterial and antifungal activity against *Escherichia coli* [6,7], *Staphylococcus aureus* [8], *Listeria monocytogenes* [9,10], *Pseudomonas aeruginosa* [11,12], *Aspergillus flavus* [13], *Fusarium sambucinum* [14], and *Candida* spp. [15]. Moreover, CIN has been demonstrated to be a potent anti-mycotoxigenic compound, reducing fungal biomass and down-regulating Aflatoxin B1 biosynthetic gene expression [16,17].

Vanillin (4-hydroxy-3-methoxybenzaldehyde, VN), the main component of vanilla beans, is a phenolic aldehyde commonly used as a flavoring agent. Plenty of studies have demonstrated the antibacterial and antifungal activity of VN against *E. coli* [6], *Salmonella enteriditis, Listeria innocua* [18], *P. aeruginosa* [19], *Saccharomyces cerevisiae* [20], and *A. flavus* [21]. 

These antimicrobial agents have variable efficiency since they must be applied in low concentrations to avoid affecting the sensory characteristics of the food. Furthermore, they are hydrophobic molecules, which favors their efficacy as they enable the interaction with the cell membrane of the microorganism. However, it is problematic in many food matrixes because the essential oil partitions into the lipid phase, whereas bacteria thrive in the aqueous phase, which could reduce the antimicrobial effect [22,23,24]. A strategy to solve these drawbacks is encapsulation, which allows lipophilic components to be encapsulated and protected from adverse environmental conditions while favoring their incorporation into high-moisture foods [25,26].

One of the most important advantages of encapsulation is the controlled, sustained, or targeted release of the active agent [27]. Emulsions can significantly improve microbial growth inhibition by slowing the release rate of the active component. Prolonging the releasing time produces a stronger inhibition through the interfacial film effect, where the antimicrobial concentration in the aqueous phase increases in partition equilibrium with the oil phase [28,29]. Therefore, the encapsulation of hydrophobic antimicrobials improves their dispersion in the aqueous phase, favoring the contact and interactions between the hydrophobic compound and microbial cell membranes [30]. Thus, the controlled release maximizes the biological activity, generating long-term inhibitory effects and extending food shelf life [31].

Different studies have shown that encapsulation could increase the antimicrobial activity of essential oils. In this sense, Sharma et al. (2022) [32] demonstrated that a nanoemulsion of clove oil with whey protein concentrate (WPC) decreased the minimum inhibitory concentration (MIC) by half for both *E. coli* and *Bacillus subtilis* [32]. At the same time, Zhang et al. (2016b) verified that the encapsulation of thyme oil with WPC increased the antimicrobial efficiency of the oil against *E. coli*, *S. enterica*, and *L. monocytogenes* in melons [33]. Nevertheless, there are also reports of studies where this increase in antimicrobial activity did not occur. Such is the case of Mauriello et al. (2021) who evaluated the encapsulation of carvacrol with whey protein isolate (WPI), in which a decrease in the antimicrobial efficiency of the oil on *Pseudomonas fluorecens*, *Staphylococcus epidermidis*, and *S. cerevisiae* was observed [34].

Therefore, the main objective of this work was to study the effect of the encapsulation of cinnamaldehyde and vanillin with WPC, and to assess the potential increase in antimicrobial activity compared to the unencapsulated antimicrobial. Hence, microencapsulated systems were formulated and their effectiveness against three model microorganisms (Gram-negative *E. coli*, Gram-positive *Listeria innocua*, and the yeast *S. cerevisiae*) in a high-humidity food (whey protein beverage) was evaluated. In addition, the effect of some combined treatments with both encapsulated systems was evaluated to assess a possible additive or synergistic effect. Finally, the microbial response was quantified and analyzed in terms of the parameters of the Gompertz and Weibull mathematical models.

## 2. Materials and Methods

### 2.1. Materials

Vanillin (MW 152.15 g/mol) and cinnamaldehyde (MW 132.16 g/mol) were purchased as a pure substance (99%) (Sigma Aldrich, Saint Louis, MO, USA). The whey protein concentrate (WPC) was acquired from Prinal (Lactoprin 80, Prinal, Santiago, Chile). According to the manufacturer, the proximal composition of WPC was 78.6% whey protein, 5.3% fat, 6.0% moisture, and 4.0% ash. Ethanol was purchased from Winkler (Santiago, Chile), and the culture media tryptone soy agar (TSA) and potato dextrose agar (PDA) were purchased from Merck (Darmstadt, Germany).

### 2.2. Preparation of Vanillin Solution

A vanillin solution (20% *w*/*v*) was prepared in an ethanol–water mixture (4:6 ratio). The solution of vanillin was dissolved by heating at 60 °C for 15 min and cooled.

### 2.3. Encapsulation

Encapsulated systems of the natural antimicrobials cinnamaldehyde (E-CIN) and vanillin (E-VN) were prepared using whey protein concentrate (WPC) as the encapsulating agent. The WPC/antimicrobial proportions were chosen according to a previous work as follows, respectively: 1:1 for E-CIN and 2:1 for E-VN [35]. The methodology for the encapsulation was described in Sepúlveda et al. (2024) [35]. Briefly, the appropriate amount of WPC (5 or 10 g) was hydrated in distilled water under stirring at 4 °C overnight. For the E-CIN, cinnamaldehyde (5% *w*/*w*) was added to the protein suspension and the emulsion was formed by high shear homogenization at 15,000 rpm for 5 min using a homogenizer (D-160, DLAB Scientific Co., Beijing, China). For the E-VN, vanillin solution (5% *w*/*w*) was added to the hydrated protein suspension and homogenized using the same protocol described above for E-CIN. The encapsulated systems were fractionated in amber Eppendorf tubes (2 mL) and stored at −18 °C for subsequent experiments. The E-CIN and E-VN were characterized by measuring the droplet size distribution, which was determined by laser diffraction using a particle size analyzer (Partica LA960, Horiba, Kyoto, Japan).

### 2.4. Preparation of the Whey Protein Beverage

The whey protein beverage was prepared according to a previous work [35] by hydrating WPC in sterile water under agitation at 4 °C overnight. Then, it was mixed with 30% clarified apple juice (L’Onda, Lima, Peru), obtaining a beverage with 4% protein. Stevia (Daily, Santiago, Chile) was added as a sweetener, and pH 4 was adjusted with citric acid. This beverage was analyzed for titratable acidity and soluble solids content. The titratable acidity was determined by the method described in Wu et al. (2020) [36]. The total soluble solids content (°Brix) was determined with a refractometer (Hanna Instruments Inc., Woonsocket, RI, USA). 

### 2.5. Microbial Inactivation

The effectiveness of the natural antimicrobials CIN and VN, as well as their respective encapsulated systems E-CIN and E-VN, and their combinations (E-CIN + E- VN) was evaluated on three microorganisms, *Escherichia coli*, *Listeria innocua*, and *Saccharomyces cerevisiae*, following the methodology of Sepúlveda et al. (2024) [29]. The free antimicrobial or the appropriate volume of the respective encapsulated system was added to the beverage under constant stirring, reaching 0.75 g/L or 1.0 g/L for cinnamaldehyde, and 1.0 g/L or 1.5 g/L for vanillin. It is worth mentioning that *L. innocua* was only evaluated with 0.75 g/L of CIN and E-CIN, 1.5 g/L of VN and E-VN, and the combined treatments because the rest of the treatments were reported in a previous study [29]. Each microorganism (100 μL) was inoculated to reach a final inoculum density of approximately 5 × 10^5^ CFU/mL. Once inoculated, a sample was taken (t = 0 day) and the respective microbiological count was performed. The untreated control was the inoculated beverage with no addition of antimicrobials. The inoculated beverages were stored at 4 °C in the dark for 14 days, and microbial counts were performed every 48 h.

### 2.6. Enumeration of Survival Cells 

Surviving *E. coli* and *L. innocua* cells were enumerated by surface plating using TSA, incubating at 37 °C for 24 h and 48 h, respectively. Similarly, *S. cerevisiae* cells were enumerated using PDA and incubated at 28 °C for 48 h. The curves of the surviving microorganisms were plotted as a function of time. Each condition was evaluated in triplicate, in independent trials.

### 2.7. Modeling the Survival Curves

Survival curves were generated by plotting log N_t_/N_0_ versus treatment time. The following mathematical models were applied to quantify the microbial response, aiming to compare the effectiveness of the treatments.

#### 2.7.1. Modified Gompertz Model

The modified version of the general Gompertz equation [37] was fitted to the survival curves:

(1) $$\log\left(\frac{N_{t}}{N_{0}}\right)=Ce^{-e(A+Bt)}-Ce^{-e(A)}$$

where the estimated parameters (*A*, *B*, and *C*) represent the different regions of the survival curve: *A* is the initial shoulder [min], *B* is the maximum death rate [min^−1^], and *C* the overall change in the number of survivors [-].

#### 2.7.2. Weibull Model

The survival curves were analyzed by fitting the cumulative form of the Weibull-type frequency distribution of resistances model, as described by Peleg and Cole (1998) [38].

(2) $$S\left(t\right)=\log\left(\frac{N_{t}}{N_{0}}\right)=-b\times t^{n}$$

where *S* is the surviving fraction of microorganisms, *t* is the reaction time, and *b* and *n* are the scale and shape parameters, respectively. The parameter *b* in the Weibull function represents the degree of inactivation of microbial cells, while the parameter *n* indicates the concavity or the trend of the survival curve. The *b* and *n* values were used to obtain the frequency distribution of the resistance curves with the following equation:

(3) $$\frac{d\phi }{dt_{c}}=bnt_{c}^{n-1}exp(-bt_{c}^{n})$$

where *t*_*c*_ is a measure of the resistance or sensitivity of the microorganism, and $\frac{d\phi }{dt_{c}}$ is the Weibull distribution corresponding to *t*_*c*_. Other statistical parameters (distribution mode: *t*_*cm*_; mean: $\bar{t_{c}}$; variance: *σ*_*tc*_^2^; skewness: *v*_1_) were calculated with the equations reported by Peleg and Cole (1998) to better explain the observed frequencies. The mode of the distribution *t*_*cm*_ represents the treatment time at which most of the population dies or is inactivated. The average $\bar{t_{c}}$ corresponds to the average inactivation time with its respective variance *σ*_*tc*_^2^. The skewness coefficient *v*_1_ represents the lack of symmetry of the distribution.

### 2.8. Statistical Analysis

The model parameters were obtained through nonlinear regression techniques. The models were internally validated using the adjusted coefficient of determination (R^2^_adj_) and Fisher’s test. All regression analyses were applied using Statgraphics Centurion XV.II^®^ (StatPoint Technologies Inc., Warrenton, VA, USA).

## 3. Results and Discussion

### 3.1. Characterization of the Whey Protein Beverage and the Encapsulated Systems

The whey protein beverage had the following characteristics: pH of 5.2 ± 0.1 and titratable acidity of 0.20 ± 0.03 g/mL malic acid from the apple juice. The total soluble solids were 6.8 ± 0.3 °Brix provided by the sugars from the apple juice (mainly fructose and, to a lesser extent, glucose and sucrose).

The encapsulated cinnamaldehyde (E-CIN) presented a droplet size in the range of 0.12–115 μm with a median of 2.2 ± 0.1 μm, while the encapsulated vanillin (E-VN) had a particle size range of 0.08–60 μm with a median of 0.2 ± 0.1 μm. Therefore, both encapsulated systems were classified as microparticles. The full information on the droplet size distribution can be seen in Sepúlveda et al. (2024) [35].

### 3.2. Microbial Inactivation

The effect of the natural antimicrobials cinnamaldehyde, vanillin, and their respective encapsulated systems (E-CIN and E-VN) was assessed against three different types of model microorganisms inoculated into the whey protein beverage. Gram-negative *E. coli* and Gram-positive *Listeria innocua* were selected because these bacteria are subrogates of pathogens (*E. coli* O157:H7 and *L. monocytogenes*) that have caused outbreaks related to dairy products. Additionally, some studies have reported that encapsulated essential oils present different effectiveness in inhibiting Gram-positive or Gram-negative bacteria. The effect on *Saccharomyces cerevisiae* was also evaluated as a model yeast that frequently deteriorates fruit juices such as apple juice.

**Figure 1:**
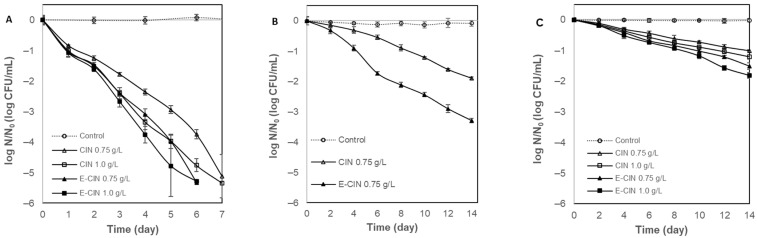
Evaluation of cinnamaldehyde (CIN) and encapsulated cinnamaldehyde (E-CIN) in inoculated whey protein beverages. (**A**) *Saccharomyces cerevisiae;* (**B**) *Listeria innocua*; (**C**) *Escherichia coli*.

Figure 1 shows that cinnamaldehyde was effective at inactivating all the microorganisms with different levels of inactivation. Among them, *S. cerevisiae* was the most sensitive, with an inactivation of more than 5 log cycles in all CIN treatments, whether with or without encapsulation, after 7 days at 4 °C (Figure 1A). The unemulsified CIN 0.75 g/L showed a 1.9 log CFU/mL reduction in *L. innocua* (Figure 1B). However, *E. coli* proved to be the most resistant microorganism to cinnamaldehyde, as it only reduced the bacterial count by 1.0 and 1.2 log CFU/mL for CIN 0.75 and 1.0 g/L, respectively (Figure 1C). These results are in line with some studies that reported that encapsulated essential oils have been significantly more effective in inhibiting Gram-positive bacteria than Gram-negative bacteria. One possible explanation is that, due to differences in the cell walls, Gram-negative bacteria present a lipopolysaccharide outer membrane that protects them from damage caused by various inhibitors [30].

The great sensitivity of *S. cerevisiae* against CIN was already observed in laboratory media by Liu et al. (2021) [1]. These authors used CIN 0.25 g/L, obtaining a reduction of 6 log cycles after 40 h of storage at 35 °C [1]. On the other hand, a reduction in *L. monocytogenes* by 3.9 log CFU/mL was reported in a model system with CIN 1.0 g/L during storage at 4 °C [9]. Additionally, the minimum inhibitory concentration (MIC) of CIN described in laboratory media for *E. coli* was between 0.31 and 0.45 g/L [6,39]. However, in this study, a higher concentration (CIN 1.0 g/L) added to the protein beverage only reduced *E. coli* by 1.2 log CFU/mL in 14 days of refrigerated storage. This shows that the food matrix considerably affects the minimum effective concentration of the antimicrobial for growth inhibition, making it necessary to increase the MIC three to four times to obtain the same effect in food matrices [40].

In this sense, Baskaran et al. (2010) reported that CIN~0.8 g/L in apple juice and apple cider presented a reduction in *E. coli* O157:H7 by 5 log cycles after 5 days of storage at 4 °C [41]. This greater inactivation may be due to several factors, such as the more acidic pH (pH 3.6 for cider and 3.8 for juice), the strain used, and the absence of whey proteins. 

Encapsulation has significantly enhanced the antimicrobial activity of cinnamaldehyde (E-CIN). The most remarkable impact was seen on *L. innocua*, whose inactivation increased by 1.4 log cycles, resulting in a reduction of 3.3 log CFU/mL when using 0.75 g/L E-CIN for 14 days at 4 °C (Figure 1B). E-CIN also proved to be more effective against *E. coli*, although to a lesser extent (between 0.5 and 0.6 log cycles), reaching 1.5 and 1.8 log CFU/mL using E-CIN 0.75 and 1.0 g/L, respectively (Figure 1C). Regarding *S. cerevisiae*, it was the most sensitive microorganism to unemulsified cinnamaldehyde with an inactivation greater than 5 log CFU/mL, so the encapsulation did not produce an additional degree of inactivation (Figure 1A).

The antibacterial effects of CIN-Tween^®^ 20 emulsions (CIN-TW 0.8 wt%, 2.4 wt%, and 4.0 wt%) in watermelon juice were evaluated on *E. coli*, *S.* Typhimurium, and *S. aureus* over 48 h by Jo et al. (2015) [36]. They found that the CIN-TW 4.0 wt% effectively controlled the growth of the three microorganisms with respect to the control. The antimicrobial effect against *S*. Typhimurium increased with the CIN-TW concentration during the incubation period. However, the growth of *E. coli* and *S. aureus* exhibited no significant differences with the different concentrations of CIN-TW [42]. 

Similar results were reported by Zhang et al. (2023) [17], who demonstrated that the encapsulation of cinnamaldehyde with alginate increases the antifungal capacity compared to the free CIN by studying the fungal growth on peanut pods during 4 months of storage [17]. They concluded that the superior performance of encapsulated CIN over free CIN was related to the controlled release capacity, resulting in the prolonged inhibitory effect during long-term storage. One of the reasons that supported it was that, due to encapsulation, the oxidative stability of the antimicrobial agent increased, which has already been verified in other studies with essential oils, such as lemon oil [43]. Additionally, Krogsgård-Nielsen et al. (2016) [22] reported that encapsulation enhanced the efficacy of isoeugenol against Gram-positive and Gram-negative bacteria in laboratory media and carrot juice, but not in milk [22].

Notwithstanding, other studies have reported that such an increase in the antimicrobial effect of the encapsulated systems was not always observed. Liao et al. (2021) observed no apparent increase in the reduction in *E. coli* or *L. innocua* when encapsulating cinnamaldehyde with soy lecithin or caseinate [44]. Other reports showed no differences between the free compound and its emulsion, such as in the emulsification of lemon myrtle oil with Tween 80 [45] or the emulsification of carvacrol with medium-chain triglycerides and Tween 80 [46]. Other studies in which cinnamaldehyde was emulsified with medium-chain triglycerides and Tween 80 demonstrated that the minimum inhibitory concentration of *E. coli* for both free and emulsified cinnamaldehyde was the same. However, when the effect was measured over time, a greater antimicrobial activity of the emulsified compound was observed [47].

**Figure 2:**
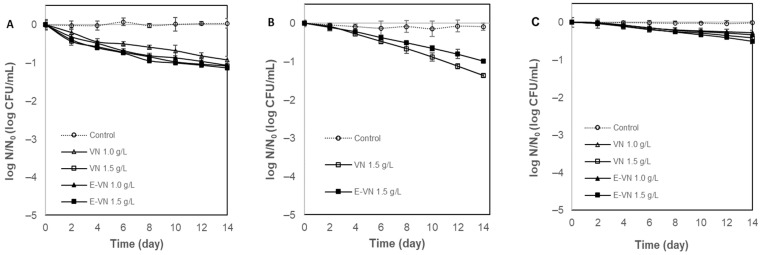
Evaluation of vanillin (VN) and encapsulated vanillin (E-VN) in inoculated whey protein beverages. (**A**) *Saccharomyces cerevisiae*; (**B**) *Listeria innocua*; (**C**) *Escherichia coli*.

Regarding vanillin treatment (Figure 2), *S. cerevisiae* was notably more resistant to vanillin than to cinnamaldehyde. There were also no significant differences between the vanillin treatment and the corresponding encapsulated system on the yeast (Figure 2A). Vanillin (1.0 and 1.5 g/L) reduced *S. cerevisiae* by 0.9 and 1.0 log CFU/mL in the protein beverage, while the E-VN 1.0 and 1.5 g/L caused a reduction of 1.0–1.1 log CFU/mL after 14 days of storage (Figure 2A). This result is similar to that reported by Fitzgerald et al. (2004) for *S. cerevisiae* in apple juice with vanillin (1.5 g/L) stored at 8 °C for 14 days [48]. This low effect of vanillin was also reported by other authors who consider that VA has a bacteriostatic but not bactericidal effect, which contrasts with the most powerful phenolic antimicrobials such as eugenol, carvacrol, and thymol, which have a bactericidal action [5].

*L. innocua* was the most sensitive microorganism, decreasing its population by 1.4 log CFU/mL with VN 1.5 g/L (Figure 2B). No increase in the antimicrobial activity of vanillin was observed with encapsulation. As with cinnamaldehyde, *E. coli* was the most resistant microorganism. The reduction in this microorganism was minimal, since 1.0 g/L and 1.5 g/L of vanillin in the protein beverage reduced by 0.3 and 0.4 log cycles, while the encapsulated vanillin (E-VN) did not significantly improve this inactivation (Figure 2C).

The MIC of vanillin in semi-skimmed milk has been reported to be 1.0 g/L and 2.5 g/L for *E. coli* O157:7 and *L. monocytogenes*, respectively [49]. Contrary to what was observed in this study, *L. monocytogenes* turned out to be more resistant than *E. coli* O157:H7. These authors also carried out a storage study of semi-skimmed milk with different concentrations of vanillin. They observed that 1.5 g/L VN reduced *E. coli* O157:H7 by ~1.5 log cycles, while that same concentration of the antimicrobial was ineffective in the inactivation of *L. monocytogenes* after 14 days at 7 °C. Furthermore, they demonstrated that the vanillin antimicrobial effect was bacteriostatic rather than bactericidal, and it was dependent on time, temperature, culture medium, and the target microorganism [49].

Regarding the combinations of the encapsulated antimicrobials, mixes of the two lower concentrations (E-CIN 0.75 g/L + E-VN 1.0 g/L) and the two higher concentrations (E-CIN 1.0 g/L + E-VN 1.5 g/L) were assessed to evaluate a possible additive or synergistic effect with the least modification in the flavor of the beverage. The effect of the combination of two antimicrobial compounds is considered synergistic when the antimicrobial activity is greater than the effect of the sum of the individual components. An additive effect is obtained when the combination of antimicrobials has an effect equal to the sum of the individual compounds. Finally, antagonism occurs when a mix of antimicrobial compounds has a combined effect less than when applied separately [18,50].

**Figure 3:**
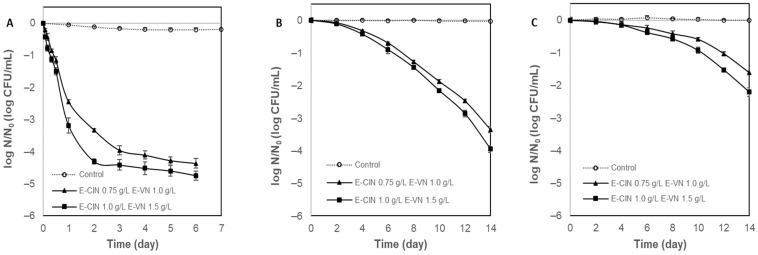
Evaluation of combined treatments with encapsulated cinnamaldehyde and vanillin (E-CIN + E-VN) in inoculated whey protein beverages. (**A**) *Saccharomyces cerevisiae*; (**B**) *Listeria innocua*; (**C**) *Escherichia coli*.

The combinations tested were most effective against *S. cerevisiae*, with rapid inactivation in the first 3 days of storage (Figure 3A). A synergism against the yeast was observed in both evaluated combinations (E-CIN 0.75 g/L + E-VN 1.0 g/L and E-CIN 1.0 g/L + E-VN 1.5 g/L), since after 3 days of storage, they already produced an inactivation of 4.0 and 4.4 log cycles, respectively, thus exceeding the sum of each single effect.

The combination of the encapsulated antimicrobials had an additive effect on *E. coli*, resulting in a reduction of up to 2.2 log CFU/mL as shown in Figure 3C. Additionally, the mixture reduced *L. innocua* by 3.4 and 4.0 log CFU/mL (Figure 3B). However, on this microorganism, when compared with the effectiveness of the single encapsulated cinnamaldehyde, the addition of E-VN resulted in an increase in the antimicrobial activity, leading to an additional reduction between 0.1 to 0.5 log cycles (considering the information of the previous work for CIN 1.0 g/L [29]). Therefore, the combination had an increased antimicrobial effect on *L. innocua*, but it did not reach the sum of the activities of both single systems.

No studies measuring the combined activity of cinnamaldehyde and vanillin in dairy products have been found. However, there is a study using vanillin in combination with extracts of cinnamon bark and cinnamon leaves in laboratory medium [6]. They observed an additive effect between the oleic cinnamon extracts and vanillin on the reduction in *L. monocytogenes*, while for *E. coli* O157:H7, a synergistic effect was described. This differs from the results obtained in this work, where a synergistic effect was observed only against *S. cerevisiae*.

Both cinnamaldehyde and vanillin are phenylpropanoids that get their name from the aromatic phenol group and the three-carbon propene tail of cinnamic acid, used in the biosynthesis. Hyldgaard et al. (2012) [50] attributed the mode of action of cinnamaldehyde to the reactive aldehyde groups, which could cross-link covalently with DNA and proteins through amine groups interfering with their normal function. They mention that at low concentrations, cinnamaldehyde inhibits different cytokinesis enzymes, affecting bacteria cell separation although septa are present after division. At higher but sub-lethal concentrations, cinnamaldehyde gains access to the periplasm and inhibits the activity of transmembrane ATPase, while at lethal concentrations, they suggest that cinnamaldehyde interacts with the cell membrane, but it is not yet clear how it perturbs the cell membranes. One possibility is altering the membrane lipid profile. Regarding yeasts, they consider that the primary mode of action is the inhibition of cell division by affecting the cell wall-synthesizing enzymes [50].

On the other hand, it was demonstrated that vanillin disrupted the membrane integrity of bacterial cells, although it may also have intracellular target sites [50,51]. The mechanism of vanillin’s antifungal activity is unclear, but it has been suggested that the aldehyde moiety of vanillin plays an important role in its antifungal activity. This is based on the observations that some yeasts can convert sublethal concentrations of vanillin into non-inhibitory compounds. Such is the study in which *S. cerevisiae* converted vanillin into vanillic acid and vanillyl alcohol, which do not possess antimicrobial activity, confirming the key role of the aldehyde fraction [50,51].

### 3.3. Modeling the Survival Curves

#### 3.3.1. Modified Gompertz Model

Since most inactivation curves of *S. cerevisiae*, *L. innocua,* and *E. coli* in the whey-apple juice beverage added with the encapsulated antimicrobials notoriously deviated from linearity, kinetic data were quantified by means of a nonlinear model like the modified Gompertz equation. The model was applied to the survival curves, explaining over 93% of the observed variation in the experimental data since the adjusted determination coefficients (R^2^_adj_) varied between 93.4 and 99.9% (Table 1, Table 2 and Table 3). Cinnamaldehyde encapsulation notoriously increased *L. innocua* inactivation, as represented by an increase in the death rate (parameter *B*) and global inactivation (parameter *C*) (in terms of absolute value), and a decrease in the shoulder parameter (*A*), indicating greater effectiveness of the antimicrobial agent when it was encapsulated. Meanwhile, on *E. coli* and *S. cerevisiae*, the death rate and global inactivation were increased when using the E-CIN; however, no defined trend in the shoulder parameter was observed (Table 1).

Regarding vanillin encapsulation, Gompertz parameters confirmed that there was no additional increase in the antimicrobial effectiveness of encapsulated vanillin on the three microorganisms evaluated, with a slight variation in the kinetic parameters between free and encapsulated vanillin (Table 2).

The Gompertz model applied to the combined treatments E-CIN + E-VN in both evaluated concentrations clearly highlighted the synergistic effect of the combination on *S. cerevisiae*, where a large increase in the death rate was observed (*B*), along with a decrease in the shoulder parameter (*A*) (Table 3). This led to the inactivation of the microorganisms in half the time, compared to the addition of individual E-CIN, demonstrating the synergistic effect of the combination of encapsulated antimicrobials.

The combination of E-CIN + E-VN on *E. coli* whose effect was described as additive showed an increase (in absolute value) in the Gompertz parameters with respect to the individual treatments, leading to a greater logarithmic reduction in microorganisms (*C*) at a greater inactivation rate (*B*) (Table 3). However, there was also an increase in the shoulder parameter (*A*), indicating an increase in the lag phase time. Hence, inactivation was slower at the beginning but reached a greater global inactivation. A similar trend was observed in the kinetic parameters of *L. innocua*. However, the increase in the total log reduction (*C*) did not reach the sum of the effects of the individual antimicrobials. Therefore, although the effect increased with the combination of encapsulated antimicrobials, it did not become additive.

Some studies have applied the modified Gompertz model to describe the effect of combined treatments. One of them was the combination of chitosan and ultrasound against *S. cerevisiae* [52], in which the model explained over 98% of the experimental data. Likewise, it was observed that the combined treatment presented a decrease in parameter *A*, along with an increase in parameters *C* and *B*, concluding that the combination of treatments was more effective than incubation with chitosan alone. The Gompertz model was also applied to quantify the effect of the combination of UV-C light with mild thermal treatments against *E. coli*, *S. cerevisiae*, and *P. fluorescens* in carrot juices [53]. The authors observed that the increase in the temperature in the combined treatment exerted a greater inhibitory effect, leading to an increase in parameters *B* and *C*, thus explaining the greater effectiveness of the combined treatment. 

#### 3.3.2. Weibull Model

Additionally, the Weibull model was applied to the experimental data to compare the effectiveness of the treatments from a different point of view. This model considers that the entire microbial population is not equally resistant to the proposed treatment, so each individual organism is deactivated at a specific time. Therefore, the observed survival curve is the cumulative form of the corresponding temporal distribution, thus generating a spectrum of resistances, which is explained through the frequency distribution of resistances [38,53].

The Weibull equation explained over 90% of the experimental data since the adjusted coefficient of determination (R^2^_adj_) presented values between 91.9 and 99.9% (Table 4, Table 5 and Table 6). The Weibull model describes the data with the parameters *b* and *n.* Parameter *b* is the scale parameter that accounts for the magnitude of microbial inactivation, and parameter *n* is the shape or trend parameter of the survival curve.

In the treatments with cinnamaldehyde, parameter *b* increased with the encapsulation of the antimicrobial, indicating a greater degree of inactivation at a higher concentration of E-CIN, especially against *L. innocua* and *S. cerevisiae* (Table 4). The greatest effect of encapsulated cinnamaldehyde was observed on *L. innocua*, in which *b* increased more than 4 times, from 0.05–0.08 to 0.28–0.34, respectively. Furthermore, a strong change was observed in the shape parameter, which went from *n* > 1 (curves with downward concavity) to *n* < 1 (upward concavity), which is consistent with the greater effectiveness of the treatments with E-CIN. The *b* and *n* parameters were used to generate the frequency distribution of resistances and the associated statistics: mean, mode, variance, and skewness (Table 4). The frequency statistics demonstrated the higher susceptibility of *L. innocua* to the encapsulated antimicrobial, changing from a wide distribution of death times with CIN to narrower distributions with a lower mode, mean, and variance when E-CIN was used (Table 4).

A similar trend was observed on *S. cerevisiae* treated with E-CIN, which showed an increase in the degree of inactivation (parameter *b*) (Table 4). The resistance frequency curves presented narrower curves and a lower mean and variance than the CIN curves. Therefore, most of the population was inactivated at shorter times with E-CIN. Nonetheless, the encapsulation of cinnamaldehyde did not markedly improve the antimicrobial activity on *E. coli.* The values of *b* and *n* did not undergo major changes, although the mean and variance of the frequency curves decreased (Table 4).

Regarding vanillin, scarce variation in the Weibull parameters confirmed that the encapsulation did not improve the antimicrobial activity with respect to free vanillin (Table 5). The statistics of the frequency distributions showed large values of mean and variance, demonstrating that the Weibull model has some limitations when the microbial inactivation is low.

The application of the Weibull model to the combined treatments (E-CIN + E-VN) clearly reflected the behavior of the respective survival curves (Figure 3). The highest degree of inactivation occurred for *S. cerevisiae,* which presented the highest value of parameter *b* (2.52 and 2.02 for E-CIN 0.75 + E-VN 1.0 and E-CIN 1.0 + E-VN 1.5 g/L, respectively) and the lowest values of the shape parameter (*n* < 1) with upward concavity (Table 6), whereas *E. coli* and *L. innocua* had a lower degree of inactivation and *n* > 1, which led to curves with downward concavity (Table 6). 

**Figure 4:**
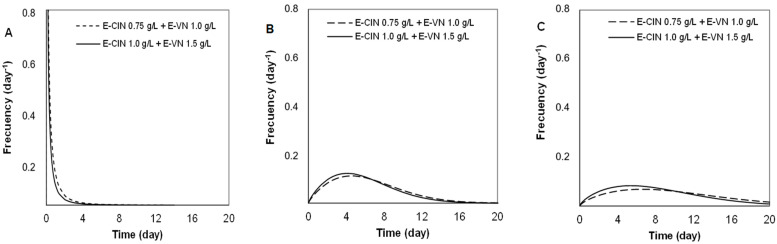
Frequency distributions of combined treatments with encapsulated cinnamaldehyde and vanillin (E-CIN + E-VN) in inoculated whey protein beverages. (**A**) *Saccharomyces cerevisiae*; (**B**) *Listeria innocua*; (**C**) *Escherichia coli*.

Likewise, the frequency curves explained the marked inactivation of *S. cerevisiae* in a very short time, with the narrowest curves, strongly skewed to the right, showing that most of the population was sensitive to the mix of encapsulated antimicrobials at low contact times and presented the lowest values of mode, mean, and variance (Figure 4A; Table 6). The frequency distributions of *L. innocua* and *E. coli* as affected by the combined treatments (Figure 4B,C) were markedly flat, leading to an increase in the mode and mean values with a higher variance. This behavior implied great heterogeneity of the response, with a significant spread of inactivation times within the population and, thus, a poorer efficacy of the treatment on the bacteria.

In general, both models managed to largely explain the experimental results. However, each mathematical model has its limitations; thus, using them simultaneously complements them. The modified Gompertz model was more versatile in fitting different shapes of survival curves, being useful in resolving the poor fit of the Weibull model in the case of biphasic curves. Such is the case of the combined treatments E-CIN 1.0 g/L + E-VN 1.5 g/L against *S. cerevisiae*, where a curve with no shoulder and tailing region was obtained (R^2^_adj_ 91.9%), as well as for curves with a prolonged shoulder and subsequent accelerated inactivation, such as the combined treatments E-CIN 0.75 g/L + E-VN 1.0 g/L on *E. coli* (R^2^_adj_ 93.6%).

These results are in line with the analysis suggested by Peleg (2024), who compared different microbial inactivation kinetics models for different shapes of survival curves and highlighted that the Gompertz model has a unique advantage over the other survival models with its applicability to scenarios where there is substantial residual survival even after long exposure to the lethal agent [54].

In agreement with these results, García-Carrillo et al. (2017) [55] reported a similar behavior in modeling survival curves of *E. coli* and *S. cerevisiae* subjected to UV-C light treatment combined with mild thermal treatments (40–50 °C) in carrot-orange juices, where it was observed that the fit of the Gompertz model was more appropriate than the Weibull model, especially for the fit of curves with an extended shoulder or tailing region. On the other hand, the authors stated that the Weibull model was more appropriate in cases where the Gompertz model overestimated the value of parameter *C* [55].

## 4. Conclusions

Encapsulation with whey protein proved to be a good strategy to enhance the antimicrobial activity of cinnamaldehyde, which might allow its future implementation as an antimicrobial agent in whey protein beverages. In contrast, vanillin encapsulation did not improve its antimicrobial activity. Further research is necessary to find an adequate formulation for the encapsulation of vanillin, thus enhancing its antimicrobial activity to be able to use this compound as an antimicrobial.

Both the modified Gompertz and Weibull mathematical models described the effect of the encapsulation of both antimicrobials. The modified Gompertz model provided kinetic parameters to better explain the differences between the free antimicrobials and their encapsulated versions, while the Weibull model allowed a better understanding of the effect of the encapsulated antimicrobials on the microbial population through the frequency distributions.

## Figures and Tables

**Table 1:** Estimated Gompertz kinetic parameters for the treatments with cinnamaldehyde (CIN) and encapsulated cinnamaldehyde (E-CIN).

Microorganism	Treatment (g/L)	*C*	*A*	*B*	VE% (R^2^_adj_)	Fisher
*S. cerevisiae*	CIN 0.75	−6.28	1.24	−0.36	93.4	65 *
	CIN 1.0	−6.86	1.03	−0.35	98.1	238 **
	E-CIN 0.75	−6.16	1.42	−0.47	93.3	64 *
	E-CIN 1.0	−7.05	1.19	−0.44	99.8	2074 **
*L. innocua*	CIN 0.75	−3.71	1.31	−0.13	99.9	3458 **
	E-CIN 0.75	−4.13	0.97	−0.20	99.1	490 **
*E. coli*	CIN 0.75	−1.97	0.60	−0.11	99.8	2027 **
	CIN 1.0	−2.30	0.49	−0.11	99.8	2744 **
	E-CIN 0.75	−2.09	0.89	−0.16	98.5	300 **
	E-CIN 1.0	−2.96	0.93	−0.13	98.4	272 **

* Statistically significant at the 0.05% level; ** statistically significant at the 0.01% level; Gompertz parameters: *A*, shoulder; *B*, death rate; and *C*, overall inactivation. VE, variability explained.

**Table 2:** Estimated Gompertz kinetic parameters for the treatments with vanillin (VN) and encapsulated vanillin (E-VN).

Microorganism	Treatments (g/L)	*C*	*A*	*B*	VE% (R^2^_adj._)	Fisher
*S. cerevisiae*	VN 1.0	−1.25	0.37	−0.21	95.7	143 *
	VN 1.5	−1.48	0.24	−0.29	96.4	207 *
	E-VN 1.0	−1.39	0.38	−0.26	98.4	417 **
	E-VN 1.5	−1.45	0.39	−0.30	98.2	409 **
*L. innocua*	VN 1.5	−1.69	1.49	−0.21	98.8	330 **
	E-VN 1.5	−1.48	1.06	−0.14	99.1	457 **
*E. coli*	VN 1.0	−0.29	1.59	−0.34	99.5	996 **
	VN 1.5	−0.69	0.72	−0.13	99.2	553 **
	E-VN 1.0	−0.41	1.12	−0.20	99.7	1296 **
	E-VN 1.5	−0.95	1.10	−0.12	99.3	560 **

* Statistically significant at the 0.05% level; ** statistically significant at the 0.01% level; Gompertz parameters: *A*, shoulder; *B*, death rate; and *C*, overall inactivation. VE, variability explained.

**Table 3:** Estimated Gompertz kinetic parameters for the combined encapsulated treatments (E-CIN + E-VN).

Microorganism	Treatment (g/L)	*C*	*A*	*B*	VE% (R^2^_adj_)	Fisher
*S. cerevisiae*	E-CIN 0.75 + E-VN 1.0	−6.43	0.09	−1.07	99.7	1020 **
	E-CIN 1.0 + E-VN 1.5	−6.08	0.35	−1.59	99.4	688 **
*L. innocua*	E-CIN 0.75 + E-VN 1.0	−4.90	1.83	−0.19	99.0	324 **
	E-CIN 1.0 + E-VN 1.5	−5.16	1.82	−0.19	97.0	104 *
*E. coli*	E-CIN 0.75 + E-VN 1.0	−3.11	2.27	−0.18	96.5	77 *
	E-CIN 1.0 + E-VN 1.5	−4.92	2.03	−0.16	98.8	230 **

* Statistically significant at the 0.05% level; ** statistically significant at the 0.01% level; Gompertz parameters: *A*, shoulder; *B*, death rate; and *C*, overall inactivation. VE, variability explained.

**Table 4:** Estimated Weibull parameters for the treatments with cinnamaldehyde (CIN) and encapsulated cinnamaldehyde (E-CIN).

Microorganism	Treatment (g/L)	*b* (day^−n^)	*n*	VE% (R^2^_adj_)	Fisher	Mode (day)	Mean (day)	Variance (day^2^)	Skewness (-)
*S. cerevisiae*	CIN 0.75	0.48	1.17	97.5	144 *	0.37	1.75	2.2	1.8
	CIN 1.0	0.68	1.04	99.6	872 *	0.07	1.43	1.9	2.0
	E-CIN 0.75	0.54	1.24	98.1	187 *	0.43	1.53	1.5	1.8
	E-CIN 1.0	0.78	1.09	99.1	377 *	0.12	1.22	1.3	2.0
*L. innocua*	CIN 0.75	0.05	1.41	99.8	1352 *	3.62	7.98	33.1	1.6
	E-CIN 0.75	0.28	0.94	98.4	247 *	0.20	3.96	17.8	2.3
*E. coli*	CIN 0.75	0.08	0.99	99.7	1275 *	0.16	13.81	195.5	2.1
	CIN 1.0	0.10	0.94	99.7	1283 *	0.62	11.64	154.2	2.3
	E-CIN 0.75	0.11	1.00	99.5	722 *	0.00	9.51	90.3	2.1
	E-CIN 1.0	0.10	1.10	98.6	640 *	0.95	7.88	51.2	1.9

* Statistically significant at the 0.01% level. Weibull parameters: *b*, magnitude of microbial inactivation; *n*, shape parameter. VE, variability explained.

**Table 5:** Estimated Weibull parameters for the treatments with vanillin (VN) and encapsulated vanillin (E-VN).

Microorganism	Treatment	*b* (day^−n^)	*n*	VE% (R^2^_adj._)	Fisher	Mode (day)	Mean (day)	Variance (day^2^)	Skewness (-)
*S. cerevisiae*	VN 1.0	0.15	0.68	98.6	361 **	5.45	21.31	1048.5	3.5
	VN 1.5	0.30	0.49	99.7	1852 **	12.74	24.10	3123.1	6.5
	E-VN 1.0	0.23	0.59	99.4	983 **	6.79	19.14	1196.4	4.4
	E-VN 1.5	0.30	0.51	99.1	658 **	9.43	19.91	1846.7	5.8
*L. innocua*	VN 1.5	0.046	1.28	99.9	4737 **	3.38	10.15	63.5	1.7
	E-VN 1.5	0.046	1.16	99.9	5261 **	2.57	13.63	139.3	1.9
*E. coli*	VN 1.0	0.028	0.90	95.8	91 *	4.63	56.05	3889.3	2.4
	VN 1.5	0.030	1.00	99.2	447 **	0.01	33.81	1144.0	2.1
	E-VN 1.0	0.024	1.01	98.9	334 **	0.40	40.76	1630.1	2.1
	E-VN 1.5	0.019	1.25	99.5	618 **	6.65	22.46	327.1	1.7

* Statistically significant at the 0.05% level; ** statistically significant at the 0.01% level. Weibull parameters: *b*, magnitude of microbial inactivation; *n*, shape parameter. VE, variability explained.

**Table 6:** Estimated Weibull parameters for the combined encapsulated treatments (E-CIN + E-VN).

Microorganism	Treatment (g/L)	*b* (day^−n^)	*n*	VE% (R^2^_adj_)	Fisher	Mode (day)	Mean (day)	Variance (day^2^)	Skewness (-)
*S. cerevisiae*	E-CIN 0.75 + E-VN 1.0	2.02	0.49	94.9	60 **	0.26	0.49	1.3	6.4
	E-CIN 1.0 + E-VN 1.5	2.52	0.41	91.9	42 **	0.25	0.32	0.9	9.7
*L. innocua*	E-CIN 0.75 + E-VN 1.0	0.029	1.79	99.9	2029 ***	4.54	6.35	13.4	1.4
	E-CIN 1.0 + E-VN 1.5	0.036	1.78	99.9	2273 ***	4.09	5.80	11.4	1.4
*E. coli*	E-CIN 0.75 + E-VN 1.0	0.017	1.68	93.6	34 *	6.68	10.25	39.5	1.4
	E-CIN 1.0 + E-VN 1.5	0.024	1.67	95.7	53 **	5.36	8.29	26.1	1.4

* Statistically significant at the 0.05% level; ** statistically significant at the 0.01% level; *** statistically significant at the 0.001% level. Weibull parameters: *b*, magnitude of microbial inactivation; *n*, shape parameter. VE, variability explained.

## Data Availability

The original contributions presented in the study are included in the article, further inquiries can be directed to the corresponding author.
